# ClinGen Bayesian-Framework-Guided Interpretation of Compound Heterozygous *F12* Variants in a Pregnant Woman with Factor XII Deficiency: A Case Report

**DOI:** 10.3390/diagnostics16081180

**Published:** 2026-04-16

**Authors:** Kyung Sun Park, Ha-eun Cho

**Affiliations:** Department of Laboratory Medicine, Kyung Hee University College of Medicine, Kyung Hee University Hospital, Kyung Hee University Medical Center, Seoul 02447, Republic of Korea; ha-eun0111@naver.com

**Keywords:** *F12* gene, Factor XII deficiency, isolated aPTT prolongation, clinical exome sequencing, ClinGen Bayesian framework, variant interpretation

## Abstract

**Background and Clinical Significance:** Isolated prolongation of activated partial thromboplastin time (aPTT) without a bleeding tendency presents a frequent diagnostic challenge and often leads to prolonged, inconclusive evaluations. **Case Presentation:** We report the case of a pregnant woman with long-standing isolated aPTT prolongation in whom clinical exome sequencing enabled a definitive diagnosis. Two compound heterozygous variants in *F12* were identified: NM_000505.4:c.1561G>A, p.(Glu521Lys), previously reported in Factor XII deficiency, and a novel in-frame insertion, NM_000505.4:c.1423_1425dup, p.(Cys475dup), absent from population databases and the prior literature. Familial genetic testing confirmed a trans configuration. Factor XII activity was markedly reduced to 1%, and mixing studies showed complete correction, consistent with coagulation factor deficiency without inhibitors. Variant interpretation using ClinGen specifications within a Bayesian framework classified both variants as likely pathogenic. Despite significant laboratory abnormalities, the patient experienced no bleeding or thrombotic complications and underwent cesarean delivery without adverse events. **Conclusions:** This case highlights that early integration of next-generation sequencing and quantitative variant interpretation frameworks can facilitate timely diagnosis, clarify clinical significance, and support appropriate management in patients with unexplained isolated aPTT prolongation.

## 1. Introduction

The activated partial thromboplastin time (aPTT) is a fundamental screening test for evaluating abnormalities in the intrinsic and common coagulation pathways. Although aPTT prolongation is frequently encountered in clinical practice and may arise from diverse causes, markedly prolonged aPTT is often identified incidentally in patients without clinically significant bleeding [1,2]. This presents a recurring diagnostic dilemma and frequently leads to prolonged and inconclusive evaluations.

Isolated aPTT prolongation that corrects on mixing and is not associated with bleeding can be diagnostically challenging [2,3]. In some patients, even after routine intrinsic pathway factor assays and inhibitor studies, a clear cause cannot be identified. In this setting, deficiencies in contact system factors, including Factor XII, prekallikrein, and high-molecular-weight kininogen, should be considered, as they are well-recognized causes of this laboratory pattern. Among these, Factor XII deficiency is classically associated with marked aPTT prolongation in the absence of clinically significant bleeding, such that aPTT is a poor predictor of hemorrhagic risk in this disorder [4,5,6].

Although Factor XII deficiency has long been considered a benign laboratory abnormality, its broad clinical implications remain unclear. Conflicting reports have suggested associations with thrombosis, recurrent pregnancy loss, or angioedema, implicating Factor XII in biological pathways beyond hemostasis [7,8,9,10,11,12]. However, these associations have been inconsistently observed, and their clinical relevance remains unclear. This uncertainty is particularly problematic in high-risk settings, such as pregnancy, surgery, or delivery, where unexplained coagulation abnormalities may prompt unnecessary concern or intervention.

The rapid expansion of next-generation sequencing (NGS) has reshaped diagnostic strategies in laboratory medicine. Genetic testing is increasingly used not only to confirm suspected diagnoses but also to elucidate the underlying cause of unexplained laboratory abnormalities. In patients with persistent isolated aPTT prolongation, early application of NGS offers an opportunity to bypass prolonged laboratory-driven investigations and directly identify genetic etiologies [2].

However, the widespread use of NGS has introduced new challenges for the interpretation of variants. Although the 2015 American College of Medical Genetics and Genomics and the Association for Molecular Pathology standards and guidelines (ACMG/AMP) [13] and subsequent ClinGen specifications (https://clinicalgenome.org/, accessed on 20 February 2026) have established a standardized framework for classification, interpretation remains particularly difficult for rare autosomal recessive disorders and novel variants, especially in-frame insertions or deletions, for which well-calibrated computational criteria are limited. Although ideal, time-sensitive clinical contexts, such as functional validation studies, are often impractical, necessitating reliance on integrated genetic, laboratory, and phenotypic evidence.

Factor XII deficiency provides an instructive model for understanding clinical and interpretative challenges in modern genomic diagnostics. Here, we report the case of a pregnant woman with long-standing isolated aPTT prolongation in whom clinical exome sequencing identified compound heterozygous variants in *F12*, including a novel in-frame insertion. By applying the 2015 ACMG/AMP [13] and ClinGen sequence variant interpretation (SVI) criteria within a Bayesian framework, we demonstrated how structured variant interpretation can resolve diagnostic uncertainty, inform clinical management, and prevent unnecessary interventions in conditions traditionally regarded as clinically benign.

## 2. Case Presentation

### 2.1. Genetic Analysis and Interpretation

Ethical approval was obtained from the Institutional Review Board (IRB) of the Kyung Hee University Medical Center (No. 2026-01-092). Clinical exome sequencing was performed in a patient with prolonged aPTT. Genetic variant interpretation was based on the 2015 ACMG/AMP [13] and ClinGen specifications (https://clinicalgenome.org/, accessed on 20 February 2026).

Briefly, population allele frequency was assessed using the Genome Aggregation Database (gnomAD v4.1.0; https://gnomad.broadinstitute.org/, accessed on 20 February 2026). Evolutionary conservation was evaluated using PhyloP scores [14]. In silico predictive evidence was obtained using BayesDel, MutPred2, REVEL, VEST4, AlphaMissense, ESM1b, and VARITY_R for missense variants [15,16]. In-frame insertion or deletion variants (INDELs) were estimated using PROVEAN, SIFT-indel, MutPred-indel, and MutationTaster [17,18,19,20]. For the PM3 code, targeted Sanger sequencing of the trio family members was performed, and we applied the ClinGen recommendations for the trans criterion PM3 (https://clinicalgenome.org/tools/clingen-variant-classification-guidance/, accessed on 20 February 2026). InterPro (https://www.ebi.ac.uk/interpro/, accessed on 20 February 2026) and UniProt (https://www.uniprot.org/, accessed on 20 February 2026) were used to identify functional domains.

### 2.2. Case Description

A 21-year-old woman was found to have prolonged aPTT incidentally during hospitalization for infectious colitis and upper respiratory infections. Coagulation testing revealed an activated partial aPTT of 106 s, with a normal prothrombin time (PT) of 13.5 s (Table 1). In a 1:1 mixing study with normal pooled plasma, aPTT was corrected to 39 s, within the reference range, indicating a factor deficiency rather than an inhibitor. Her complete blood count (CBC) was unremarkable, with a white blood cell (WBC) count of 6500/µL, hemoglobin of 12.0 g/dL, and platelet count of 203,000/µL. Factor VIII activity was 76%, which was within the reference range; thus, the most common form of hemophilia A was considered unlikely. She reported that her wounds tended to heal slowly and that she bruised somewhat more easily than others; however, she never experienced spontaneous bleeding, menorrhagia, or excessive bleeding after dental procedures or trauma. Because she experienced no significant symptoms or disability, she did not return for follow-up at that time.

Fourteen years later, she became pregnant with her first child and presented to our hospital after routine prenatal laboratory tests revealed markedly prolonged aPTT. At this visit, CBC values remained normal (WBC 7120/µL, hemoglobin 12.3 g/dL, platelet count 250,000/µL). Coagulation testing showed an aPTT of 157.9 s and a PT of 13.0 s. The fibrinogen level was 302 mg/dL and the D-dimer 0.27 µg/mL. Factors VIII and IX activities were 124% and 83%, respectively, and inhibitors of both factors were negative. Targeted Sanger sequencing of F8 and F9 revealed no pathogenic or likely pathogenic variants. Based on normal intrinsic factor levels and negative F8/F9 sequencing, it was highly unlikely that she was affected by or was a carrier of hemophilia A or B, although she was advised to be observed carefully for excessive bleeding around the time of delivery.

### 2.3. Genetic Diagnosis

**Figure 1:**
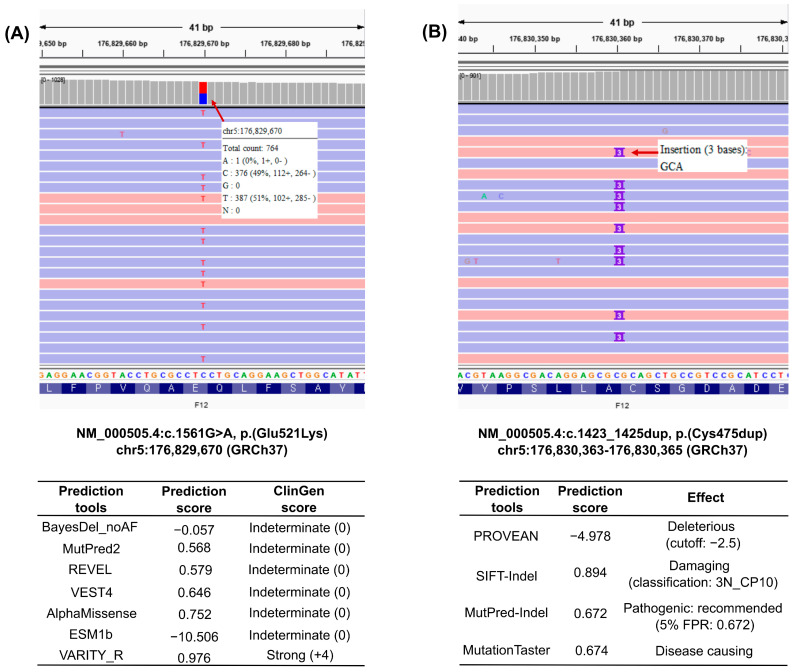
**Identification and computational interpretation of compound heterozygous F12 variants.** (**A**) Integrative Genomics Viewer (IGV) screenshot showing the heterozygous missense variant, NM_000505.4:c.1561G>A, p.(Glu521Lys), in *F12* (Chr5:176829670, GRCh37). The variant was detected by clinical exome sequencing with a sequencing depth of 752× and a variant allele fraction (VAF) of 0.51, consistent with heterozygosity. In silico prediction results are summarized below the IGV panel. Among seven calibrated computational tools, only VARITY_R provided strong pathogenic evidence (ClinGen score +4), whereas the remaining tools yielded indeterminate results. (**B**) IGV screenshot showing the heterozygous in-frame duplication, NM_000505.4:c.1423_1425dup, p.(Cys475dup), in *F12* (Chr5:176830363–176830365, GRCh37). The variant was supported by a sequencing depth of 714× and a VAF of 0.51, also consistent with heterozygosity. Multiple in-frame indel–specific prediction tools (PROVEAN, SIFT-indel, MutPred-Indel, and MutationTaster) consistently predicted a deleterious effect.

Clinical exome sequencing (CES) was then performed. CES identified two heterozygous variants in *F12*: NM_000505.4:c.1561G>A (p.(Glu521Lys), chr5:177402669 (GRCh38), Chr5:176829670 (GRCh37)) and NM_000505.4:c.1423_1425dup (p.(Cys475dup), chr5:177403362-177403364 (GRCh38), Chr5:176830363-176830365 (GRCh37)) (Figure 1). The c.1561G>A, p.(Glu521Lys) variant is rare (global population allele frequency, 6.2 × 10^−6^) in the general population (gnomAD v4.1.0, https://gnomad.broadinstitute.org/, accessed on 20 February 2026). A review of previously reported cases identified this variant in the homozygous state in two individuals with Factor XII deficiency, one of Chinese origin [21] and one of Taiwanese [22] origin [22]. In both patients, the aPTT was markedly prolonged to greater than 100 s, and both Factor XII coagulant activity (FXII:C) and Factor XII antigen levels (FXII:Ag) were profoundly reduced. Functional studies using transient expression of wild-type and mutant FXII in HEK293T cells demonstrated a significant functional reduction in mutant FXII expression compared with the wild-type. In silico pathogenicity prediction using seven computational tools showed strong evidence of pathogenicity (ClinGen point: +4) only from VARITY_R, whereas the remaining tools (BayesDel, MutPred2, REVEL, VEST4, AlphaMissense, and EsM1b) yielded indeterminate results (ClinGen point: 0) without evidence of pathogenicity.

Taken together, the rarity of the variant in the general population, the highly specific and consistent clinical and laboratory phenotypes observed in previously reported homozygous patients, in vitro functional assay data demonstrating impaired FXII function, and the genotype-phenotype correlation support the classification of the c.1561G>A, p.(Glu521Lys) variant as likely pathogenic according to the 2015 ACMG/AMP [13] and ClinGen SVI guidelines (https://clinicalgenome.org/, accessed 20 February 2026), based on the evidence codes PM2_Supporting (+1), PP4 (+1), PM3 (+2), and PS3_Moderate (+2). Using the Bayesian framework for variant interpretation [23,24], these evidence weights yielded a total score of +6, which independently supported the classification of likely pathogenic. Notably, inclusion of computational evidence (PP3) would have increased the cumulative Bayesian score, resulting in a range of +6 to +10 depending on the weighting applied. However, given the substantial variability in pathogenicity predictions across in silico tools—where only one tool (VARITY_R: PP3_Strong) provided strong evidence while others yielded indeterminate results—the contribution of PP3 was interpreted with caution.

**Figure 2:**
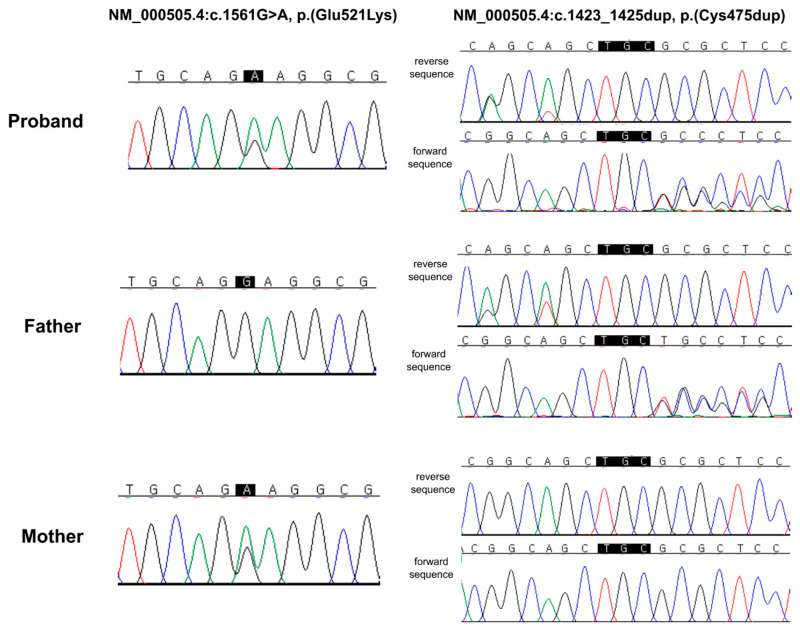
**Familial segregation analysis of the F12 variants by Sanger sequencing.** Sanger sequencing chromatograms demonstrating segregation of the two *F12* variants within the family. The proband was heterozygous for both NM_000505.4:c.1561G>A (p.(Glu521Lys)) and NM_000505.4:c.1423_1425dup (p.(Cys475dup)). The c.1561G>A variant was identified in the mother, whereas the c.1423_1425dup variant was detected in the father, confirming that the variants are present in trans in the proband.

The c.1423_1425dup, p.(Cys475dup) variant was absent from the general population database (gnomAD v4.1.0; https://gnomad.broadinstitute.org/, accessed on 20 February 2026) and has not been previously reported in the literature, representing a novel *F12* variant. To assess the pathogenicity of this in-frame insertion, multiple prediction tools, specifically designed for in-frame insertion or deletion variants, including PROVEAN, SIFT-indel, MutPred-Indel, and MutationTaster, were used [17,18,19,20]. All four tools consistently predicted deleterious effects, supporting the potential functional effect of this variant. To further interpret these findings, familial genetic testing was performed on the patient’s parents (Figure 2). The father had an aPTT of 35.7 s and a PT of 12.4 s, and the mother had an aPTT of 40.2 s and a PT of 13.7 s, all of which were within the respective reference ranges (Table 1). Neither parent had a history of abnormal bleeding. Familial genetic testing revealed that the p.(Glu521Lys) variant was present in the mother, whereas the p.(Cys475dup) variant was inherited from the father, indicating that the patient was compound heterozygous for the two *F12* variants.

Additional coagulation Factor assays were performed. Factor XI activity was mildly reduced, at 59%, whereas Factor XII activity was profoundly decreased, at 1%. However, clinical exome sequencing did not reveal any pathogenic or likely pathogenic variants of the *F11* gene. At that time, the patient’s aPTT remained markedly prolonged at 126.6 s, and a repeat mixing study again demonstrated complete correction to 35.9 s, confirming the presence of a coagulation Factor deficiency without evidence of an inhibitor.

Accordingly, considering that the c.1423_1425dup, p.(Cys475dup) variant is absent from the general population, is predicted to be deleterious by multiple in-frame indel prediction tools, is present in trans with the c.1561G>A, p.(Glu521Lys) variant previously classified as likely pathogenic, and that the patient exhibited profoundly reduced Factor XII activity, consistent with locus homogeneity in Factor XII deficiency, a clear genotype–phenotype correlation was established. Based on these findings, the c.1423_1425dup, p.(Cys475dup) variant was classified as likely pathogenic according to the 2015 ACMG/AMP and ClinGen SVI guidelines by applying the evidence codes PM2_Supporting (+1), PM4 (+2), PM3 (+2), and PP4 (+1). Consistently, the Bayesian-framework-based classification [23,24] yielded a total score of +6, further supporting the classification of likely pathogenic causes.

Based on genetic findings and coagulation profile, the patient was diagnosed with FXII deficiency. At 38 + 1 weeks of gestation, a planned cesarean section was performed under close hematological monitoring. Fresh frozen plasma (FFP) was administered perioperatively, and the aPTT was maintained at approximately 54.6–60.9 s before and after the procedure, allowing the cesarean section to be performed safely. The patient was discharged without perioperative or postpartum bleeding complications.

## 3. Discussion

In patients with markedly prolonged aPTT that corrects on mixing studies and is not accompanied by a bleeding tendency, the differential diagnosis is often narrow and clinically predictable. In such scenarios, extensive stepwise laboratory evaluations, based solely on traditional coagulation algorithms, may offer limited value. Early consideration of contact factor abnormalities, particularly Factor XII deficiency, represents a pragmatic approach, particularly when routine intrinsic Factor assays and inhibitor studies are unclear [1].

In the present case, normal Factor VIII and IX activities, complete correction of mixing studies, and the absence of bleeding manifestations excluded hemophilia A and B. Although Factor XI activity was mildly reduced, the clinical phenotype and extent of aPTT prolongation were not consistent with Factor XI deficiency. At this diagnostic juncture, further stepwise coagulation testing was unlikely. Instead, the early application of NGS enabled prompt etiological clarification by identifying compound heterozygous variants of *F12*. This case illustrates a paradigm shift in the evaluation of unexplained isolated aPTT prolongation, in which NGS serves as a central component of the diagnostic workflow rather than a late confirmatory tool. Rather than the identification of Factor XII deficiency itself, which has been previously reported using NGS, this case highlights the value of an integrated diagnostic and interpretative approach. In particular, the structured application of the ClinGen Bayesian framework enabled transparent interpretation of challenging variants, including a novel in-frame insertion, while clarifying the distinction between Mendelian loss-of-function variants and polymorphism-driven variation. This approach also facilitated translation of a genetically well-defined diagnosis into clinical decision making in a complex setting such as pregnancy.

Beyond diagnosis, this case underscores the key challenges in variant interpretation of rare autosomal recessive disorders. Although the 2015 ACMG/AMP guidelines [13] and the subsequent ClinGen specifications (https://clinicalgenome.org/, accessed on 20 February 2026) have substantially refined variant classifications, many variants, particularly novel or rare indels, are difficult to interpret using population data or computational predictions alone. The ClinGen Bayesian framework [23,24] provides a quantitative and transparent method for integrating diverse evidence types and was particularly informative in this case, where both *F12* variants posed interpretive challenges.

For the c.1561G>A, p.(Glu521Lys) variant, pathogenicity was supported by consistent phenotypes reported in homozygous patients, disease mechanisms, and functional evidence, whereas computational predictions were highly discordant. Although ClinGen SVI allows application of PP3 based on a well-calibrated tool meeting a predefined threshold, we adopted a conservative approach considering the discordant computational results and did not assign additional weight to PP3 in the final classification.

Interpretation of the novel in-frame indel c.1423_1425dup, p.(Cys475dup), further illustrates the current limitations of the computational frameworks. Although PM4 addresses protein length changes caused by in-frame insertions or deletions, no computational criterion analogous to PP3 has been specifically calibrated for indels. Consequently, careful evaluation of the domain context, predicted functional impact, allelic configuration, familial genetic testing, and phenotypic correlations is essential. In this case, the absence of the variant from the general population database, deleterious predictions from multiple indel-specific tools, trans configuration with a likely pathogenic variant, and profoundly reduced Factor XII activity collectively established a convincing genotype–phenotype correlation. Consistent application of the 2015 ACMG/AMP [13] and ClinGen SVI criteria (https://clinicalgenome.org/, accessed on 20 February 2026) combined with Bayesian scoring allowed both *F12* variants to be classified as likely pathogenic, with a total score of +6. This quantitative approach strengthened diagnostic confidence and directly informed clinical decision making.

Although Factor XII deficiency is characterized by the absence of a bleeding phenotype, its association with thrombotic complications varies. The proposed links between venous thromboembolism, arterial thrombosis, recurrent pregnancy loss, and angioedema remain inconsistent [5,6,7,8,9,10,11,12,25,26]. This inconsistency may partly reflect the heterogeneous genetic mechanisms underlying reduced FXII activity. In particular, the commonly studied promoter variant NM_000505.4(F12):c.-4T>C (rs1801020, previously 46C>T) represents a functional polymorphism rather than a pathogenic variant in a Mendelian context [7,27]. Although direct in vitro evidence for its effect on transcription is limited, multiple clinical and laboratory studies have consistently shown that this variant significantly modulates FXII activity and aPTT, typically without causing a severe reduction in FXII activity. In contrast, true loss-of-function variants in *F12* follow an autosomal recessive inheritance pattern and underlie congenital FXII deficiency as a Mendelian disorder, in which FXII activity is often markedly reduced, frequently to <10% [8,26,28]. Importantly, many previous studies have evaluated FXII deficiency in pregnancy or thrombosis without clearly distinguishing between these fundamentally different genetic categories, which might contribute to the inconsistent clinical associations reported to date.

In the present case, the patient harbored compound heterozygous loss-of-function variants consistent with a Mendelian form of FXII deficiency, allowing a genetically well-defined interpretation distinct from polymorphism-driven variation. Nevertheless, even in this context, the relationship between FXII deficiency and pregnancy-related risk remains insufficiently defined [8]. Accordingly, FXII deficiency alone should not be interpreted as a determinant of obstetric management but rather as one component within a broader clinical framework. Clinical decision making should be guided by an integrated assessment of the patient’s overall risk profile, with close peripartum surveillance and individualized management tailored to the dynamic balance between thrombotic and bleeding risks.

In the present case, no thrombotic events, bleeding complications, angioedema, or obstetric difficulties were observed despite long-term follow-up and exposure to physiological stressors, including pregnancy and cesarean delivery. The absence of these complications is clinically meaningful and supports the concept that Factor XII deficiency alone is insufficient to confer significant thrombotic or hemorrhagic risk. This unique biological feature has led to the recognition of Factor XII as an attractive therapeutic target for antithrombotic strategies. Experimental and clinical studies have demonstrated that the inhibition of Factor XII or its downstream pathway can attenuate pathological thrombosis without significantly increasing bleeding risk, supporting the concept of “bleeding-sparing” anticoagulation [29,30].

Physiological changes during pregnancy further complicate the interpretation of coagulation parameters [8,31,32]. An estrogen-mediated increase in Factor XII levels may mask the true severity of the deficiency when measurements are obtained late in gestation. Nevertheless, Factor XII activity in this patient remained profoundly reduced even during late pregnancy, supporting the diagnosis of severe congenital deficiency and reinforcing the robustness of the genetic diagnosis. Ultimately, the patient underwent a carefully planned cesarean section under close hematological monitoring without perioperative or postpartum bleeding complications, illustrating that precise laboratory interpretation combined with structured genetic diagnosis can guide safe and individualized clinical management.

## 4. Conclusions

This case demonstrates that the integration of NGS into the diagnostic evaluation of unexplained isolated aPTT prolongation can provide timely etiological clarification and prevent prolonged and inefficient laboratory investigations. In a pregnant woman with long-standing aPTT prolongation and no bleeding phenotype, clinical exome sequencing identified compound heterozygous *F12* variants, including a novel in-frame insertion, enabling a definitive diagnosis of congenital Factor XII deficiency.

The application of the 2015 ACMG/AMP [13] and ClinGen SVI criteria within a Bayesian framework (https://clinicalgenome.org/, accessed on 20 February 2026) allowed for transparent and quantitative variant interpretation, particularly in the context of rare autosomal recessive diseases and indel variants for which computational evidence remains limited. This structured approach strengthened diagnostic confidence and directly informed clinical management, including safe peripartum planning without unnecessary intervention.

## Figures and Tables

**Table 1:** Hematologic and coagulation profiles of the proband and her parents.

Parameter	Reference Range	Proband ^1^	Father	Mother
WBC (×10^3^/µL)	4.0–10.0	4.64–7.12	6.43	4.79
Hemoglobin (g/dL)	12–16	11.4–12.5	15.8	12.0
Platelet (×10^3^/µL)	150–350	198–250	187	287
Fibrinogen (mg/dL)	200–400	302	-	-
FDP (µg/mL)	<5	<2.50	-	-
D-dimer (µg/mL)	<0.5	0.27	-	-
PT (seconds)	12.5–14.7	11.9–13.8	12.4	13.7
aPTT (seconds)	29–43	106–157.9	35.7	40.2
Mixing study aPTT (seconds)	29–43	35.9–39	-	-
Factor VIII activity (%)	60–140	76–124	-	-
Factor IX activity (%)	60–140	83–107	-	-
Factor VIII inhibitor	Negative	Negative	-	-
Factor XI inhibitor	Negative	Negative	-	-
Factor XI activity (%)	60–140	59	-	-
Factor XII activity (%)	60–140	1	-	-

^1^ Values for the proband represent the range of measurements obtained during the observation period. Abbreviations: PT, prothrombin time; aPTT, activated partial thromboplastin time; FDP, fibrin degradation products.

## Data Availability

The original contributions presented in this study are included in the article. Further inquiries can be directed to the corresponding author.
